# Peroxiredoxin V Protects against UVB-Induced Damage of Keratinocytes

**DOI:** 10.3390/antiox12071435

**Published:** 2023-07-16

**Authors:** Sin Ri Kim, Ji Won Park, Byung-Hoon Lee, Kyung Min Lim, Tong-Shin Chang

**Affiliations:** 1 Graduate School of Pharmaceutical Sciences, Ewha Womans University, 52 Ewhayeodae-gil, Seodaemun-gu, Seoul 03760, Republic of Korea; sr.kim@ewhain.net (S.R.K.); kmlim@ewha.ac.kr (K.M.L.); 2 College of Pharmacy and Research Institute of Pharmaceutical Sciences, Seoul National University, 1 Gwanak-ro, Gwanak-gu, Seoul 08826, Republic of Korea; jw3611@snu.ac.kr (J.W.P.); lee@snu.ac.kr (B.-H.L.)

**Keywords:** peroxiredoxin V, ultraviolet B irradiation, keratinocytes, reactive oxygen species, mitochondrial dysfunction, apoptosis

## Abstract

Ultraviolet B (UVB) irradiation generates reactive oxygen species (ROS), which can damage exposed skin cells. Mitochondria and NADPH oxidase are the two principal producers of ROS in UVB-irradiated keratinocytes. Peroxiredoxin V (PrxV) is a mitochondrial and cytosolic cysteine-dependent peroxidase enzyme that robustly removes H_2_O_2_. We investigated PrxV’s role in protecting epidermal keratinocytes against UVB-induced ROS damage. We separated mitochondrial and cytosolic H_2_O_2_ levels from other types of ROS using fluorescent H_2_O_2_ indicators. Upon UVB irradiation, PrxV-knockdown HaCaT human keratinocytes showed higher levels of mitochondrial and cytosolic H_2_O_2_ than PrxV-expressing controls. PrxV depletion enhanced hyperoxidation-mediated inactivation of mitochondrial PrxIII and cytosolic PrxI and PrxII in UVB-irradiated keratinocytes. PrxV-depleted keratinocytes exhibited mitochondrial dysfunction and were more susceptible to apoptosis through decreased oxygen consumption rate, loss of mitochondrial membrane potential, cardiolipin oxidation, cytochrome C release, and caspase activation. Our findings show that PrxV serves to protect epidermal keratinocytes from UVB-induced damage such as mitochondrial dysfunction and apoptosis, not only by directly removing mitochondrial and cytosolic H_2_O_2_ but also by indirectly improving the catalytic activity of mitochondrial PrxIII and cytosolic PrxI and PrxII. It is possible that strengthening PrxV defenses could aid in preventing UVB-induced skin damage.

## 1. Introduction

The skin is a major interface between the body and the environment that is essential for overall homeostasis. Ultraviolet (UV) radiation from the sun can compromise the skin’s integrity. UVB radiation is one of the most hazardous environmental factors for the skin. Reactive oxygen species (ROS) have been shown to play a critical role in regulating UVB-induced reactions in skin cells [1,2]. Epidermal keratinocytes synthesize proteins and lipids to build the stratum corneum, which protects the body against infections, chemicals, and physical harm. These procedures demand lots of energy from mitochondrial oxidative phosphorylation. UV radiation-induced ROS production damages electron transport chain (ETC) complexes, slowing mitochondrial respiration in keratinocytes [3,4,5,6]. Due to inadequate electron transport in UV-irradiated keratinocytes, dysfunctional mitochondria produce more mitochondrial ROS (mtROS), creating a vicious cycle: oxidative stress from increasing mtROS generation can overwhelm cellular antioxidant defenses and affect mitochondrial components, including ETC complexes, further impairing mitochondrial respiration [3,4,5,6]. The sustained mitochondrial dysfunction and increased ROS production in UV-irradiated keratinocytes can trigger the activation of apoptotic pathways, which lead to the release of pro-apoptotic factors and eventually result in cell apoptosis. Exposure to antioxidants reduced UVB-induced mitochondrial dysfunction [4,6] and apoptosis [7,8,9,10].

Mitochondria [11,12,13,14] and NADPH oxidase (NOX) in cell membranes [9,14] are the two principal sources of ROS in UVB-irradiated keratinocytes. mtROS originate from superoxide anion (O_2_^•−^), which is produced when O_2_ is reduced by one electron. Mitochondrial complex II and III are one-electron leakage sites in UV-irradiated keratinocytes [11,13]. Of the seven NOX family members (NOX1 through NOX5, Duox1 and Duox2), UVB irradiation activates the assembly of the NOX1 complex, which is crucial for cellular ROS-mediated DNA damage, apoptosis, and cytotoxicity in keratinocytes [15,16]. Mitochondrial O_2_^•−^ can spontaneously or enzymatically be dismutated to H_2_O_2_ by superoxide dismutase 2 (SOD2) in the mitochondrial matrix or SOD1 in the intermembrane space [17,18,19]. NOX superoxide-generating complex produces extracellular O_2_^•−^, which can also be dismutated to H_2_O_2_ spontaneously or by extracellular SOD3 [20,21]. 

Uncharged H_2_O_2_ produced in these processes can diffuse into the cytosol across mitochondrial or cell membranes, raising cytosolic H_2_O_2_, whereas anionic O_2_^•−^ is not easily membrane permeable. The SOD reaction provides partial relief from oxidative stress caused by O_2_^•−^ through the production of H_2_O_2_, which is a gentle oxidant. However, H_2_O_2_ is easily converted into a more potent oxidant, hydroxyl radical (^•^OH), via the Fenton reaction [19,22,23]. Therefore, antioxidant enzymes responsible for eliminating H_2_O_2_ are required to protect keratinocytes from UVB-induced damage such as mitochondrial dysfunction and apoptosis.

The peroxiredoxin peroxidase (Prxs) family has six members in mammals that reduce H_2_O_2_ (PrxI to PrxVI) [24]. Prxs are divided into three subgroups (typical 2-Cys, atypical 2-Cys, and 1-Cys) based on the number and position of cysteine residues participating in catalysis [24]. In contrast to other 2-Cys Prxs, PrxV is a cytoprotective antioxidant that is not inactivated by hyperoxidation of the catalytic cysteine sulfhydryl (Cys-SH) to cysteine sulfenic acid (Cys-SO_2_H) [25,26,27]. Therefore, peroxidation-resistant PrxV was expected to significantly delay the peroxidative inactivation of other 2-Cys Prxs in response to various oxidative stresses. Additionally, since PrxV is widely located throughout cells in the cytoplasm, mitochondria, nucleus, and peroxisomes, it may be favorable for the effective removal of H_2_O_2_, which can accumulate at several sites throughout the cell under conditions of oxidative stress [26,28,29].

The precise protective role of PrxV in keratinocytes exposed to UVB is still not well understood. In this study, we report that PrxV serves to protect keratinocytes against UVB-induced damage, including mitochondrial dysfunction and apoptosis, by directly removing H_2_O_2_ and indirectly improving the catalytic action of 2-Cys Prxs. Strengthening PrxV defenses could aid in preventing UVB-induced skin damage.

## 2. Materials and Methods

### 2.1. Reagents and Antibodies

The following reagents were used in this study: cytochrome C (556433) and the annexin V-fluorescein isothiocyanate (FITC) apoptosis kit (556547) were purchased from BD biosciences; caspase-3 (9662), caspase-9 (9508), and poly(ADP-ribose)polymerase (PARP) (9542) were from Cell Signaling Technology; Prx-SO_2_ (ab16830), PrxI (ab15571), and β-actin (ab8226) were from Abcam; PrxV (LF-MA0002) was from Invitrogen; 10-N-nonyl-acridine orange (NAO) (A1372) and tetramethylrhodamine ethyl ester (TMRE) (T669) were from Molecular Probes; peroxy-orange-1 (PO-1) (4944) and mitochondria peroxy-yellow-1 (MitoPY) (4428) were from Tocris Biosciences; puromycin (p8833), FuGene6 (E2311), and pSUPER-puro vector were from Sigma Aldrich, Promega and OligoEngine, respectively.

### 2.2. Cell Culture

HaCaT cells (CLS, Eppelheim, Germany) were cultured in Dulbecco’s Modified Eagle Medium supplemented with 10% fetal bovine serum and 100 U/mL of penicillin and streptomycin at 37 °C in a humidified atmosphere containing 5% CO_2_.

### 2.3. Establishment of HaCaT Cells Expressing Small Hairpin RNA Targeting PrxV

The small hairpin interfering RNA oligonucleotide sequences targeting human PrxV (5′-GGAGACAGACUUAUUACUA-3′) used to construct a pSUPER siPrxV were purchased from Genotec (Daejeon, Republic of Korea), annealed, and cloned into the pSuperior-puro (pSUPER) vector (Oligoengine). HaCaT cells were transfected with pSUPER_siPrxV vectors using FuGene6 reagent (Promega, Madison, WI, USA). Following selection with 1.5 μg/mL puromycin, single clones were expanded and characterized.

### 2.4. UVB-Irradiation

HaCaT ells were irradiated with 20 mJ/cm^2^ UVB using a Bio-Sun lamp (Vilber-Lourmat, Collegien, France) with a peak output of around 312 nm. Cells were washed twice with warm phosphate-buffered saline prior to UVB irradiation, then irradiated through a thin layer of phosphate-buffered saline at 37 °C, and re-fed with their own medium. 

### 2.5. Determination of H_2_O_2_

Cells were grown to 80% confluence on glass-bottomed 35-mm culture dishes (MatTeK, Ashland, OH, USA). After stimulation, cells were washed twice with phenol red-free culture media and incubated with each indicator in phenol red-free culture media containing 1% fetal bovine serum for 20 min at 37 °C. After the media were replaced with phenol red-free culture media containing 1% fetal bovine serum, fluorescent images were obtained on a temperature-controlled stage using the LSM 880 AiryScan (Carl Zeiss, Göttingen, Germany). To detect cytoplasmic or mitochondrial H_2_O_2_, cells were incubated with PO-1 (5 μM) or MitoPY1 (10 μM), respectively. The excitation/emission wavelengths for PO-1 and MitoPY1 were 561/595, and 488/525 nm, respectively. Fluorescence intensity was measured and visualized using NIS-Elements software 3.1 (Nikon, Tokyo, Japan).

### 2.6. Flow Cytometry Analyses

Mitochondrial damage was measured in cells stained with either TMRE (50 nM) or NAO (50 nM) at 37 °C for 20 min. To analyze cell death, cells were resuspended in annexin binding buffer and labeled with annexin-V-FITC and propidium iodide (PI) at 25 °C for 15 min, according to the manufacturer’s instructions (Annexin-V-FITC and PI kits; BD Biosciences, San Jose, CA, USA). Cells were analyzed using a FACSCalibur flow cytometer (BD Biosciences) with excitation wavelength 488 nm and observation wavelength 530 nm for green fluorescence and 585 nm for red fluorescence. Relative change in fluorescence was analyzed with FlowJo software 10.9.

### 2.7. Oxygen Consumption rate (OCR) Measurement

OCR was determined using a seahorse XFe96 or XFp analyzer (Agilent Technologies, Santa Clara, CA, USA) accompanied by an Agilent Seahorse Mito Stress Test kit (Agilent Technologies) according to the manufacturer’s instructions. Key parameters of mitochondrial respiration were analyzed in cells treated with 1 μM oligomycin, 1 μM carbonyl cyanide-4-(trifluoromethoxy)phenylhydrazone (FCCP), and a mixture of 0.5 μM antimycin A/rotenone. At the end of the Seahorse assay, a protein assay was performed to normalize the OCR measurements. OCR values were normalized for the amount of cellular protein in each well.

### 2.8. Western Blotting

Cell lysates were prepared as described previously [30]. Briefly, cells were lysed in 20 mM HEPES buffer (pH 7.0) containing 150 mM NaCl, 1% Triton X-100, 2 mM EGTA, 1 mM EDTA, 20 mM β-glycerophosphate, 10% glycerol, 1 mM AEBSF, aprotinin (10 μg/mL) and leupeptin (10 μg/mL). Cell debris was removed by centrifugation at 12,500× *g* for 10 min at 4 °C. Equal amounts of cell lysates were subjected to Western blotting analysis using specific antibodies, as indicated.

### 2.9. Preparation of Mitochondrial and Cytosolic Fractions

Subcellular fractionation was performed as described previously [31]. Cells in hypotonic buffer (25 mM KCl, 2 mM MgCl_2_, 0.5 mM EDTA, 0.5 mM EGTA, 1 mM DTT, and 10 mM HEPES, pH 7.5) were left on ice for 30 min and then homogenized using a Polytron-Aggregate homogenizer (Kinematica Inc., Luzern, Switzerland) in the presence of protease inhibitors (1 mM AEBSF, 5 μg/mL aprotinin, and 5 μg/mL leupeptin). An equal volume of hypertonic buffer (500 mM sucrose, 25 mM KCl, 2 mM MgCl_2_, 0.5 mM EDTA, 0.5 mM EGTA, 1 mM DTT, and 10 mM HEPES, pH 7.5) was added to reestablish isotonicity. Nuclei and unbroken cells were pelleted twice at 750× *g* for 10 min. The supernatant was centrifuged at 15,000× *g* for 15 min to collect the heavy membrane fraction containing mitochondria. The supernatant was then centrifuged at 100,000× *g* for 60 min and the final supernatant was collected as the cytosolic fraction.

### 2.10. Statistical Analysis

All experiments were repeated at least three times. Comparisons of data between groups were performed by Student’s *t*-test. A *p*-value less than 0.05 was considered statistically significant.

## 3. Results

### 3.1. PrxV Depletion Potentiates UVB-Induced Increases in Mitochondrial and Cytoplasmic H_2_O_2_ as Well as 2-Cys Prxs Hyperoxidation in HaCaT keratinocytes

**Figure 1:**
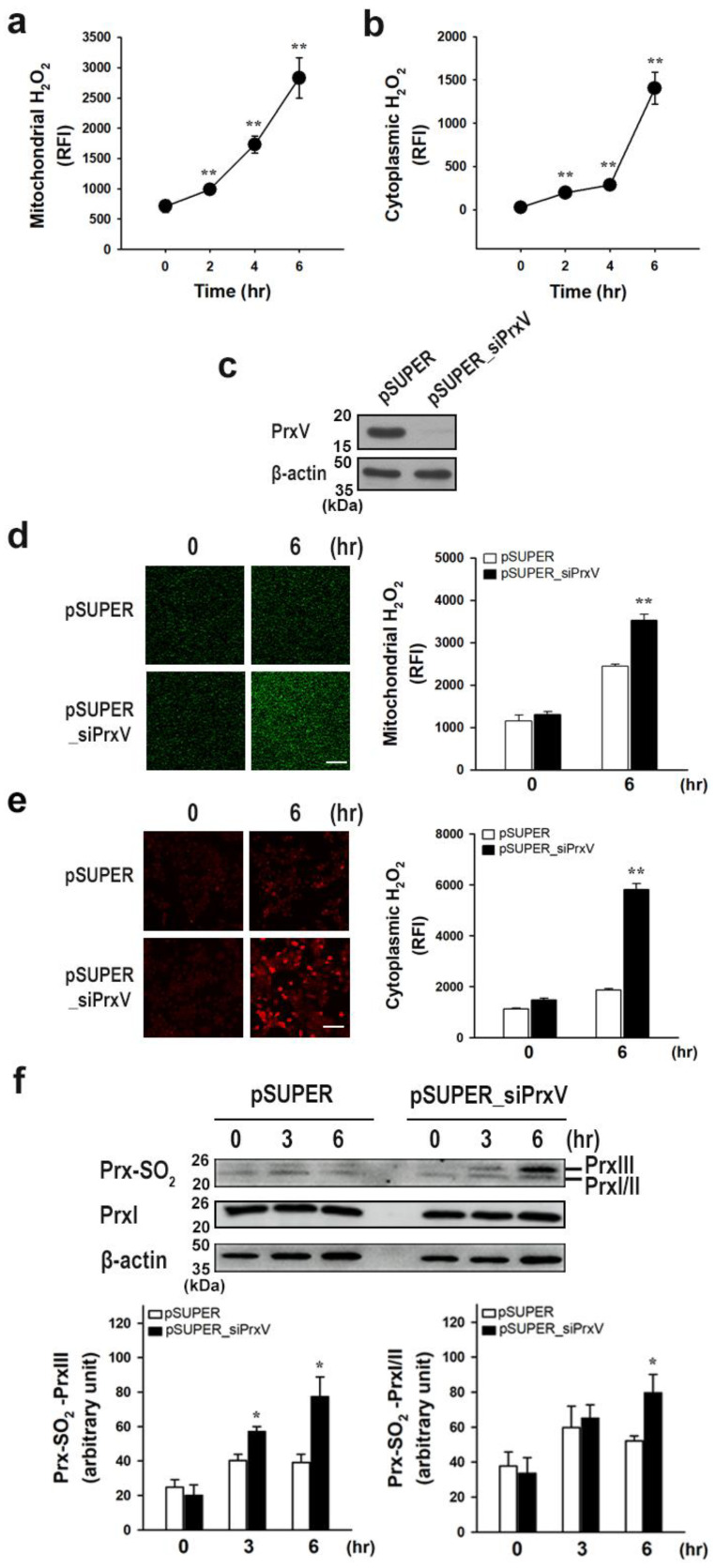
Effects of peroxiredoxin V (PrxV)-knockdown on the accumulation of H_2_O_2_ and hyperoxidation of Prxs in HaCaT keratinocytes after exposure to ultraviolet B (UVB). Normal HaCaT cells irradiated with UVB (20 mJ/cm^2^) were incubated for the indicated times and stained with MitoPY1 (**a**) or PO-1 (**b**), respectively. Fluorescence images were obtained and quantified at five regions randomly selected on each dish. Quantitative levels of mitochondrial (**a**) or cytoplasmic (**b**) H_2_O_2_ are shown as mean ± standard error of the mean (n = 5) of the RFI. ** *p* < 0.01 versus unirradiated control. (**c**) Western blotting of lysates from control (*pSUPER*) and PrxV-knockdown (*pSUPER_siPrxV)* HaCaT cells. (**d**,**e**) After being irradiated with UVB (20 mJ/cm^2^), cells were incubated for 6 h and stained with mitochondria peroxy-yellow-1 (MitoPY1) (**d**) or peroxy-orange-1 (PO-1) (**e**). Fluorescence images were quantified at five regions randomly selected on each dish. Scale bar = 100 μm. Mitochondrial (**d**) or cytoplasmic (**e**) H_2_O_2_ levels are shown quantitatively as mean ± standard error of the mean (n = 5) of the relative fluorescence intensity (*RFI*). ** *p* < 0.01 versus pSUPER. (**f**) Cells irradiated as above were incubated for the indicated times and the cell lysates were subjected to immunoblot with antibodies against Prx-SO_2_, PrxI and β-actin. Representative immunoblots are shown. The quantitative data are expressed as mean ± standard error of the mean (n = 3) of arbitrary unit. * *p* < 0.05 versus pSUPER.

HaCaT keratinocytes exposed to 20 mJ/cm^2^ UVB were probed with MitoPY1 [32] and PO-1 [33,34] fluorescent dyes, which selectively and specifically respond to mitochondrial and cytoplasmic H_2_O_2_, respectively. Cells were then visualized to investigate changes in mitochondrial and cytoplasmic H_2_O_2_. Mitochondrial and cytoplasmic H_2_O_2_ levels significantly increased over time up to 6 h after UVB irradiation (Figure 1a,b).

To determine whether PrxV protects epidermal keratinocytes from UVB-induced damage, control and PrxV-depleted stable cell lines were created. To deplete PrxV from HaCaT keratinocytes, we transfected the cells with a pSUPER_siPrxV vector, which produces small interfering RNAs specific to PrxV. Cells were transfected with a pSUPER empty vector as a control (Figure 1c shows PrxV expression). HaCaT keratinocytes stably transfected with the pSUPER and pSUPER_siPrxV vectors are hereafter referred to as pSUPER and pSUPER_siPrxV cells, respectively. PrxV depletion significantly increased mitochondrial H_2_O_2_ levels 6 h after UVB irradiation (Figure 1d). PrxV depletion also significantly increased cytoplasmic H_2_O_2_ after UVB irradiation (Figure 1e).

Elevated levels of mitochondrial and cytoplasmic H_2_O_2_ have previously been shown to enhance the hyperoxidative inactivation of mitochondrial PrxIII and cytosolic PrxI and PrxII [35]. We quantified the degree of Prx hyperoxidation using an antibody against both the sulfinic and sulfonic forms of 2-Cys Prxs [35]. After UVB exposure, increased mitochondrial and cytosolic H_2_O_2_ in pSUPER_siPrxV cells was associated with significantly increased hyperoxidation of mitochondrial PrxIII and cytosolic PrxI and PrxII compared to in pSUPER cells (Figure 1f). This suggests that PrxV contributes to the suppression of hyperoxidative inactivation of mitochondrial PrxIII and cytosolic PrxI and PrxII in keratinocytes exposed to UV light.

Together, these findings show that hyperoxidation-resistant PrxV restricts the accumulation of mitochondrial and cytosolic H_2_O_2_ in UVB-exposed keratinocytes by improving the catalytic activity of mitochondrial PrxIII and cytosolic PrxI and PrxII as well as eliminating mitochondrial and cytosolic H_2_O_2_.

### 3.2. PrxV Depletion Exacerbates UVB-Induced Mitochondrial Oxidative Damage of Keratinocytes

**Figure 2:**
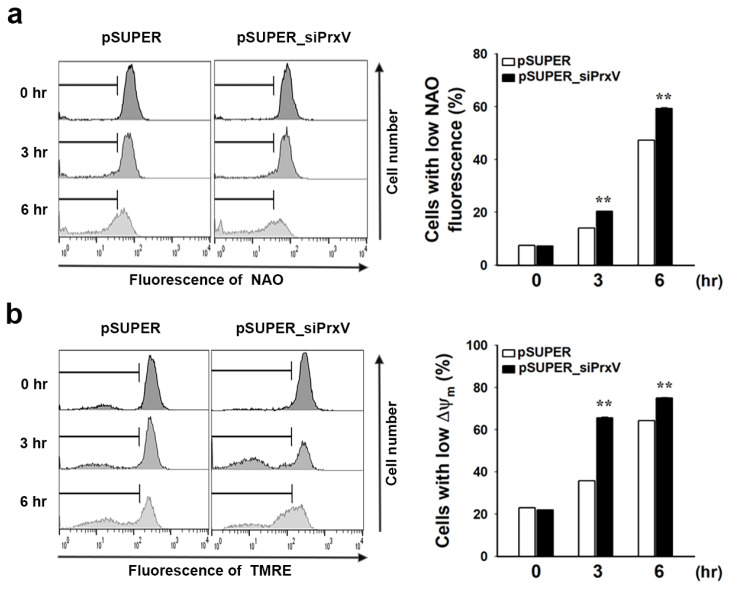
Effects of PrxV-knockdown on mitochondrial membrane potential (ΔΨm) and oxidation of cardiolipin in the mitochondrial membrane of HaCaT keratinocytes after exposure to UVB. Control (*pSUPER*) and PrxV-knockdown (*pSUPER_siPrxV)* HaCaT cells exposed to UVB (20 mJ/cm^2^) were incubated for the indicated times. The cells loaded with 10-N-nonyl-acridine orange (NAO) (**a**) or tetramethylrhodamine ethyl ester (TMRE) (**b**) were analyzed by flow cytometry. Representative histograms are shown. Quantitative data are shown as mean ± standard error of the mean (n = 5) of the percentage of cells with low NAO (**a**) or low ΔΨm fluorescence (**b**). ** *p* < 0.01 versus pSUPER.

Given that mitochondrial H_2_O_2_ accumulation has been linked to mitochondrial oxidative stress and damage [36], we investigated the oxidative modification of the mitochondrial lipid cardiolipin, an unsaturated phospholipid found in the inner mitochondrial membrane. Levels of cardiolipin oxidation were assessed using NAO, which selectively binds to mitochondrial cardiolipin and not other forms of phospholipid or oxidized cardiolipin [37,38]. Following UVB irradiation, cells exhibited decreased levels of NAO-stained mitochondria with a more pronounced effect in pSUPER_siPrxV cells than in pSUPER cells (Figure 2a). We also used TMRE to measure the change in the mitochondrial membrane potential (ΔΨm), which fluoresces in response to ΔΨm-driven mitochondrial uptake [39]. UVB-induced ΔΨm dissipation was significantly higher in pSUPER_siPrxV cells than in pSUPER cells, according to flow cytometric analysis (Figure 2b). These findings suggest that PrxV protects keratinocytes from mitochondrial oxidative damage caused by UVB irradiation.

### 3.3. PrxV Depletion Exacerbates UVB-Induced Mitochondrial Dysfunction in Keratinocytes

**Figure 3:**
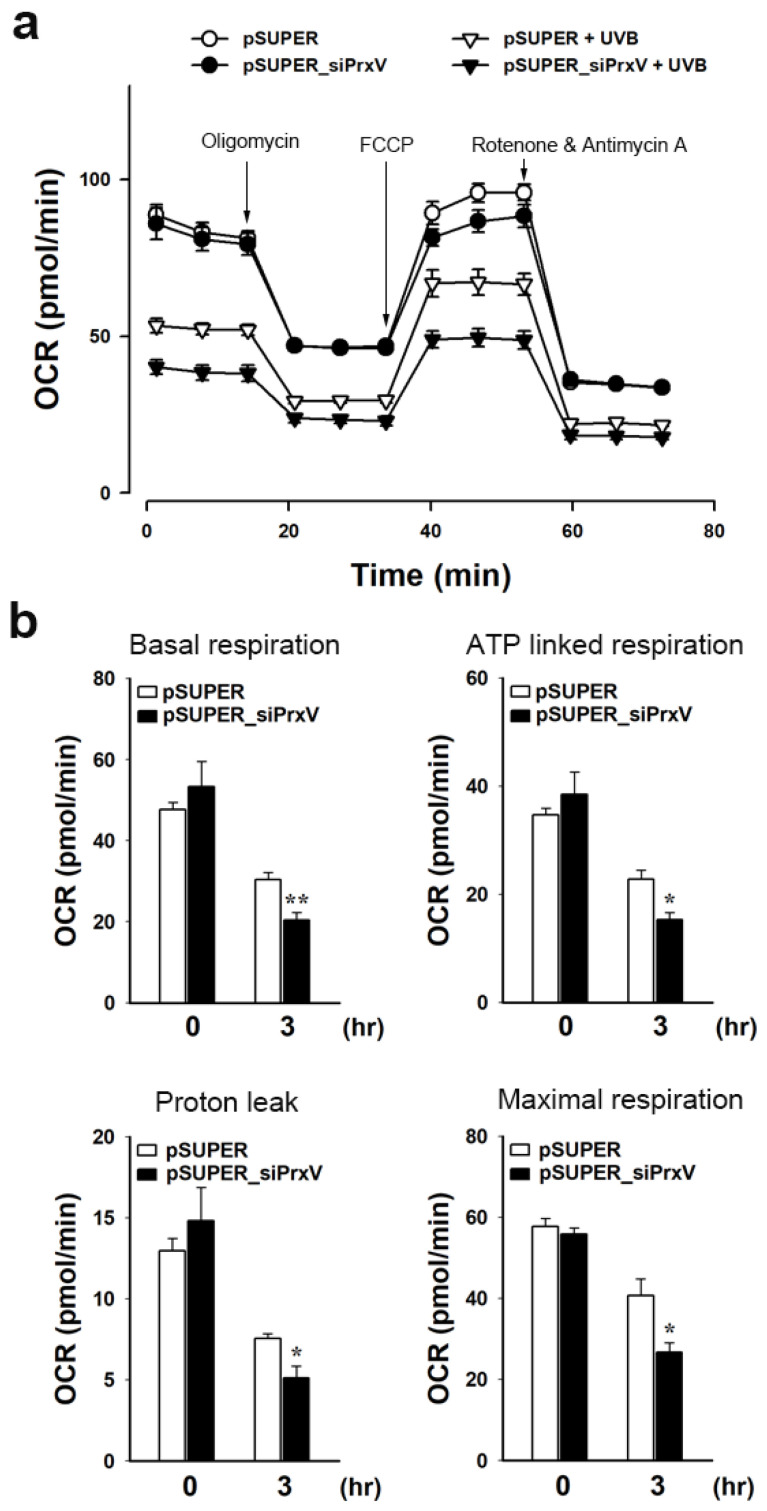
Effects of PrxV-knockdown on respiration of keratinocytes after exposure to UVB. Control (*pSUPER*) and PrxV-knockdown (*pSUPER_siPrxV)* HaCaT cells irradiated with UVB (20 mJ/cm^2^) were incubated for 3 h. (**a**) Representative tracing of oxygen consumption rate (OCR). Arrows indicate time points when cells were treated with oligomycin, carbonyl cyanide-4-(trifluoromethoxy)phenylhydrazone (FCCP), and rotenone plus antimycin A, respectively. Data are shown as mean ± standard error of the mean (n = 3). (**b**) OCR is calculated as Basal respiration, ATP-linked respiration, Proton leak, and Maximal respiration. Data are shown as mean ± standard error of the mean (n = 5). * *p* < 0.05, ** *p* < 0.01 versus pSUPER.

Because PrxV depletion worsens UVB-induced oxidative damage to a mitochondrial lipid and ΔΨm dissipation in keratinocytes (Figure 2), we examined the potential impact of PrxV-depletion on mitochondrial dysfunction following UVB exposure utilizing an extracellular flow analysis to quantify mitochondrial activity by observing OCR. Bioenergetics analysis was conducted through the addition of oligomycin, FCCP, and rotenone/antimycin A revealing altered cellular metabolic processes and OCR for HaCaT keratinocytes (Figure 3a). 

Oligomycin inhibited ATP synthase and reduced electron flow through the ETC, which led to a decrease in OCR associated with cellular ATP synthesis (Figure 3b, ATP-linked respiration). In the presence of oligomycin, the remaining oxygen consumption of mitochondria is related to the rate at which protons leak over the mitochondrial inner membrane (Figure 3b, Proton leak). The addition of the protonophore FCCP induced an artificially high proton conductance into the membrane. Thus, electron transport through the ETC was unimpeded, and oxygen consumption reached its maximal level (Figure 3b, Maximal respiration). When complexes I and III were inhibited with rotenone/antimycin A, all mitochondrial-mediated respiration was stopped, and the remaining respiration was non-mitochondrial. Basal respiration was calculated by subtracting non-mitochondrial respiration from baseline respiration (Figure 3b, Basal respiration). At 3 h following UVB exposure, there was a substantial decrease in mitochondrial respiration due to a decrease in basal and maximal respiration, which was accompanied by decreased proton leakage that affected ATP production. UVB exposure significantly lowered the mitochondrial respiration at every phase evaluated in pSUPER_siPrxV cells relative to pSUPER cells. These findings support the conclusion that PrxV protects keratinocytes from UVB-induced mitochondrial dysfunction.

### 3.4. PrxV Depletion Activates UVB-Induced Mitochondria-Mediated Apoptosis Signaling Pathways in Keratinocytes

**Figure 4:**
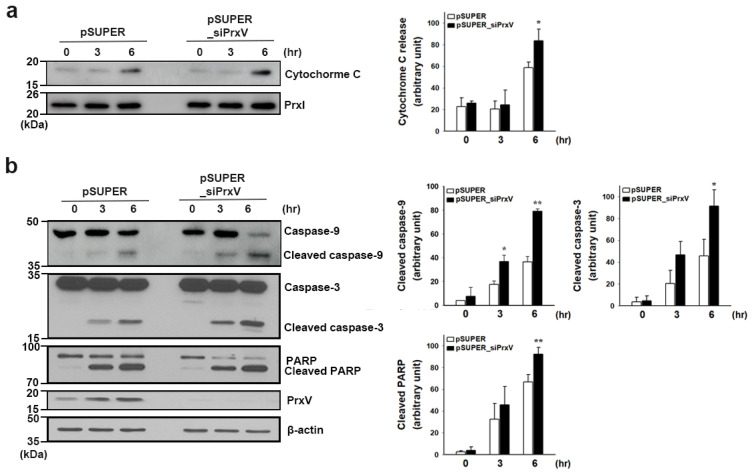
PrxV-knockdown effects mitochondria-mediated apoptosis signaling pathways in HaCaT keratinocytes after exposure to UVB. Control (*pSUPER*) and PrxV-knockdown (*pSUPER_siPrxV)* HaCaT cells were irradiated with UVB (20 mJ/cm^2^) and then incubated for the indicated times. (**a**) Cells were separated into cytosolic and mitochondrial fractions. Cytochrome c was detected in cytosolic cell fractions (marked by PrxI). Representative immunoblots are shown. The quantitative data are expressed as mean ± standard error of the mean (n = 3) of arbitrary units. * *p* < 0.05 versus pSUPER. (**b**) Western blotting for caspase-9, caspase-3, poly(ADP-ribose) polymerase (PARP), or PrxV. Representative immunoblots are shown. The quantitative data are expressed as mean ± standard error of the mean (n = 3) of arbitrary units. * *p* < 0.05, ** *p* < 0.01 versus pSUPER.

**Figure 5:**
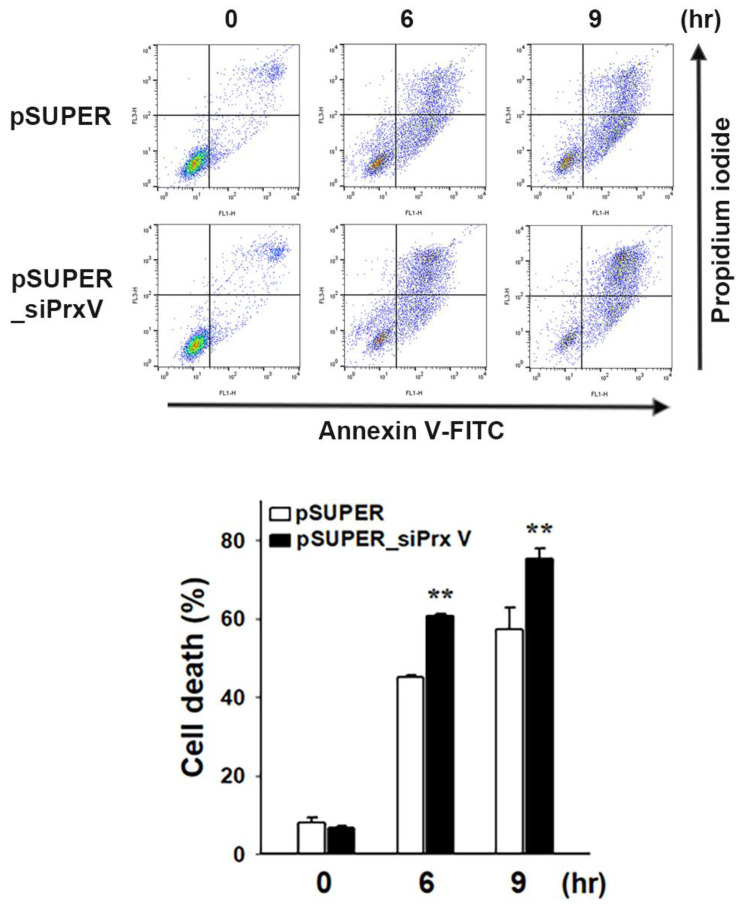
Effects of PrxV-knockdown on UVB-induced apoptosis in HaCaT keratinocytes. HaCaT cells transfected with *pSUPER* and *pSUPER_siPrxV* Control (*pSUPER*) and PrxV-knockdown (*pSUPER_siPrxV).* HaCaT cells were exposed to UVB (20 mJ/cm^2^) and incubated for the indicated times. Cell death was measured as the percentage of annexin V–fluorescein isothiocyanate (annexin V–FITC) or propidium iodide-positive cells after analysis by flow cytometry; mean ± standard error of the mean (n = 5). ** *p* < 0.01 versus pSUPER.

To determine whether the increased oxidative stress associated with the depletion of PrxV impacted cellular apoptosis, we next examined cytochrome c release from the mitochondria to the cytosol and subsequent caspase activation. The oxidation of cardiolipin, which retains cytochrome c, can occur through mtROS and increase the release of cytochrome c [40,41]. Since PrxV depletion aggravated UVB-induced oxidation of cardiolipin in keratinocyte mitochondrial membranes (Figure 2a), we examined cytochrome c release into the cytosol 6 h after UVB treatment. More cytochrome c was released into the cytosol in pSUPER_siPrxV than in pSUPER cells (Figure 4a). Immunoblot analysis revealed that PrxV depletion increased the cleavage of procaspases-3 and -9 and the caspase substrate PARP (Figure 4b). We then looked at the impact that PrxV depletion had on UVB-induced apoptotic cell death. Apoptotic cell populations were analyzed via PI and Annexin V staining and flow cytometry. UVB light exposure resulted in a time-dependent increase in apoptotic cells with significantly higher apoptosis in pSUPER_siPrxV cells compared to pSUPER cells (Figure 5). These results together indicate that PrxV suppresses mitochondria-mediated apoptosis in UVB-irradiated keratinocytes.

## 4. Discussion

**Figure 6:**
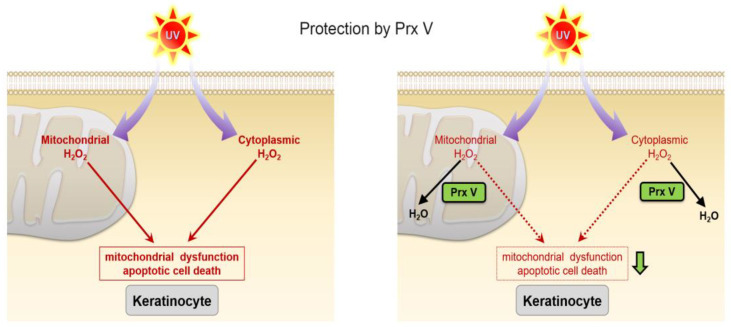
The role of PrxV in UVB-induced HaCaT cells. PrxV regulates UVB-induced mitochondrial and cytoplasmic H_2_O_2_. Decreased ROS production by PrxV prevents mitochondrial dysfunction and apoptotic cell death. PrxV prevents skin damage caused by UVB.

Together our results implicate PrxV in defense against UVB-induced keratinocyte damage through mediating activity against ROS and associated destructive pathways such as mitochondrial oxidation and apoptosis. In this study, we focused on the role of PrxV in UVB-induced HaCaT cells. We propose that PrxV prevents mitochondrial dysfunction and apoptotic cell death by decreasing mitochondrial and cytoplasmic H_2_O_2_ (Figure 6).

There are two major sources of ROS in keratinocytes irradiated with UVB: mitochondria [11,12,13,14] and NOX1 in cell membranes [9,14]. In UVB-irradiated keratinocytes, the mitochondrial complexes II and III both have the potential to cause leakage of single electrons that convert O_2_ to O_2_^•−^ [11,13]. Another mechanism of photodamage is the production of mtROS through the photosensitization of mitochondrial chromophores, which absorb UVB irradiation, resulting in unstable excited state molecules that transmit energy to neighboring O_2_, generating radicals and non-radical ROS, primarily O_2_^•−^ and H_2_O_2_ [1,42,43]. NOX1 is immediately activated in UVB-irradiated keratinocytes, though the mechanism underlying this phenomenon remains to be explained [15,16]. O_2_^•−^ mainly produced by NOX or mitochondria can be quickly dismutated to H_2_O_2_ either spontaneously or enzymatically by SODs. Whereas anionic O_2_^•−^ is not easily membrane permeable, uncharged H_2_O_2_ produced in the process can diffuse into the cytosol, raising cytoplasmic H_2_O_2_. 

Although mitochondrial O_2_^•−^ can be rapidly converted to H_2_O_2_ by SOD2 in the mitochondrial matrix [17,18,19], the overexpression of SOD2 in keratinocytes inhibited UVB-induced increase in mitochondrial O_2_^•−^ levels but had no discernible impact on apoptosis [14]. One possible explanation is that the mitochondrial oxidative stress generated by O_2_^•−^ is only partially alleviated by this SOD process and that the resulting H_2_O_2_ is a weak oxidant that readily undergoes the Fenton reaction to become the more dangerous hydroxyl radical (^•^OH) [19,22,23]. The balance between mitochondrial H_2_O_2_ production and mitochondrial H_2_O_2_ decomposing enzymes such as PrxIII and PrxV is tightly regulated [29], and glutathione peroxidase 1 and 4 [44,45]. We have previously shown that PrxIII inhibits the accumulation of mitochondrial H_2_O_2_ to protect keratinocytes from UVB-induced mtROS-mediated apoptosis [12]. However, since PrxIII readily undergoes inactivation by the hyperoxidation of its active site cysteine during catalysis [27,31], its ability to protect keratinocytes from UVB-induced apoptosis is likely to be reduced. We found that the deletion of PrxV greatly increased the hyperoxidation of PrxI, PrxII and PrxIII (Figure 1f). The GGLG motif-containing loop and the YF motif-containing C-terminal helix, according to Wood et al., are responsible for the hyperoxidation sensitization of Prxs [46]. The GGLG and YF patterns, however, are missing from human PrxV. Therefore, PrxV has been thought of as a cytoprotective antioxidant with a higher resistance to inactivation by hyperoxidation [25,26,27]. As a result, it has been predicted that PrxV could help protect keratinocytes from UVB-induced ROS-mediated damage not only by directly removing mitochondrial H_2_O_2_ but also by indirectly improving PrxIII’s catalytic action. In contrast to the other Prx family members, PrxV is present in the cytosol, mitochondria, nucleus, and peroxisomes of mammalian cells [26]. PrxV is extensively distributed throughout cells due to two translation initiation sites in PrxV mRNA and two subcellular localization signals in their translated products [47,48]. In line with the increase in cytosolic H_2_O_2_ levels in keratinocytes exposed to UVB, PrxV depletion also significantly increased the hyperoxidation of cytosolic PrxI and PrxII. 

The skin is constantly bombarded with environmental assaults, such as sunshine. UV light promotes oxidative stress and DNA damage, which accelerates the deterioration of cellular components and alters gene expression [49]. The stratum corneum serves as the main barrier against infections, chemicals, and physical damage. The production of proteins and lipids by keratinocytes to create the stratum corneum requires significant energy, which is normally produced by oxidative phosphorylation in mitochondria. Primary human epidermal keratinocytes decreased expression of several genes involved in mitochondrial function as little as 4 h after UVB exposure [6]. In particular, when mitochondrial DNA, lipid membranes, and catalytic proteins involved in energy metabolism are damaged, this can restrict the energy supply and energy-dependent repair processes [50]. Mitochondrial dysfunction in UV-irradiated keratinocytes is characterized as a decrease in the mitochondria’s ability to produce ATP due to a loss of ΔΨm, changes in ETC function, an increase in ROS production, and a decrease in oxygen consumption [3,4,5,6]. Indeed, due to their high ATP consumption and relatively poor spare respiratory capacity, HaCaT keratinocytes have been postulated to be particularly sensitive to apoptosis by insults that alter bioenergetics [51], which was supported by our results (Figure 3 and Figure 5). The vicious loop of mtROS formation, mitochondrial malfunction, and more mtROS production that eventually leads to cell apoptosis has been linked to a lower rate of mitochondrial respiration in UV-irradiated keratinocytes [3,4,5]. Similarly, this study demonstrated that PrxV depletion in keratinocytes increased H_2_O_2_ accumulation in mitochondria and cytosol after UVB exposure, which may contribute to a decrease in mitochondrial respiration and subsequently made keratinocytes more vulnerable to apoptosis via mitochondria-mediated pathways.

## 5. Conclusions

PrxV-knockdown in cultured keratinocytes increases H_2_O_2_ accumulation in mitochondria and cytosol following UVB exposure, which consequently makes keratinocytes more susceptible to apoptosis via a mitochondria-mediated route. We further found that UVB-induced mitochondrial dysfunction is enhanced in PrxV-depleted cultured keratinocytes. Our results suggest that PrxV plays a crucial role in preventing UVB-induced apoptosis and mitochondrial dysfunction in epidermal keratinocytes. PrxV not only directly removes mitochondrial and cytosolic H_2_O_2_ but also indirectly improves the catalytic activity of mitochondrial PrxIII and cytosolic PrxI and PrxII. To further support our conclusions, in future studies, we intend to further elucidate the protective role of PrxV against UVB-induced keratinocyte damage by overexpressing PrxV in keratinocytes and measuring ROS levels and damage, and by using mouse models depleted of or overexpressing PrxV. Altogether, our findings support the notion that strengthening PrxV defenses could aid in preventing UVB-induced skin damage.

## Data Availability

The data is contained within this article.
